# PD-L1 Blockade Improves Survival in Sepsis by Reversing Monocyte Dysfunction and Immune Disorder

**DOI:** 10.1007/s10753-023-01897-0

**Published:** 2023-09-30

**Authors:** Li Yang, Qian Gao, Qiujing Li, Shubin Guo

**Affiliations:** 1 Emergency Department, Beijing Shijitan Hospital, Capital Medical University, No. 10 Tieyi Road, Yangfangdian, Haidian District, Beijing, 100038 China; 2 Emergency Medicine Clinical Research Center, Beijing Key Laboratory of Cardiopulmonary Cerebral Resuscitation, Beijing Chao-Yang Hospital, Capital Medical University, No. 8, South Road of Worker’s Stadium, Chaoyang District, Beijing, 100020 China

**Keywords:** sepsis, monocyte subsets, PD-L1, cytokine

## Abstract

Monocyte dysfunction is critical to sepsis-induced immunosuppression. Programmed death ligand-1 (PD-L1) has shown a close relationship with inflammatory disorder among animal models and patients. We aimed to investigate the potential beneficial immunologic mechanisms of anti-PD-L1 on monocyte dysfunction of mice with sepsis. Firstly, we assessed the potential association between PD-L1 expression on monocyte subsets and sepsis severity as well as 28-day mortality. In this study, 52 septic patients, 28 septic shock patients, and 40 healthy controls were enrolled and their peripheral whole blood was examined by flow cytometry. Then, cecal ligation and puncture (CLP) were performed for establishing the mouse sepsis model. Subsequently, effects of anti-PD-L1 antibody on monocyte subset, major histocompatibility complex II (MHC II) expression, cytokine production, and survival were investigated. PD-L1 expression on the classical monocytes (CD14 + + CD16 −) was significantly upregulated among septic shock patients and the 28-day death group than non-septic shock group and 28-day survival group (*P* < 0.05). Compared to septic mice, anti-PD-L1-treated mice had significantly elevated percentages of major histocompatibility complex (MHC) II on peripheral Ly6c^hi^ monocyte at 24 h after CLP. Our results showed that the anti-PD-L1 antibody markedly decreased the level of serum inflammatory cytokines interleukin (IL)-6, tumor necrosis factor (TNF)-α, and IL-10 in sepsis mice at 24 h, 48 h, and 72 h, respectively (*P* < 0.05). The survival rate of CLP mice was significantly improved by anti-PD-L1 antibody treatment. Classical monocytes with high expression of PD-L1 were thought to be connected with sepsis progression. The PD-L1 blockade protects from sepsis, at least partially by inhibiting the reversal of monocyte dysfunction.

## INTRODUCTION

Sepsis is the major death cause in current global intensive care units and refers to a serious organ dysfunction induced by a deregulated host response to infection [1]. Sepsis influences nearly 30 million people all over the world per year, with a mortality rate of more than 30% [2]. Immunomodulatory disorders are the primary cause of death in patients with sepsis [3–5]. Thus, strategies to enhance host immunity need to be studied carefully and expected to be therapies for sepsis. Monocytes and macrophages form the first line of defense against pathogens [6]. Monocytes are crucial to innate and adaptive immunity by antigen presentation, cytokine secretion, and the expression of costimulatory molecules activating adaptive immunity cells. Sepsis induces monocyte activation and alters subset distribution, leading to activation of lymphocytes and adaptive immune cells, resulting in changes in plasma cytokine levels. Its manifestation was overactivated immune response to eliminate causative agents, during which process monocytes become activated and impaired in function. Thus, the attenuation of monocyte-mediated inflammatory damage provides one promising therapy for sepsis.

The programmed death 1 (PD1) receptor system is a high-efficiency immunoregulatory pathway which negatively regulates immune responses. This system is consists of PD1 and its two ligands: programmed cell death 1 ligand (PD-L1) and programmed cell death 2 ligand (PD-L2). PD-1/PD-L1 refers to a significant regulatory molecule in cell-mediated immunity and is expressed in varied immune cells, like monocytes and T lymphocytes. PD-1/PD-L1 expression is closely related to the higher risk of mortality or morbidity in animal models and patients with sepsis. PD-1/PD-L1 is viewed critical to activation and proliferation inhibition of immune cells. As seen from studies, therapy using immunomodulatory agents for boosting host immunity can elevate survival rate of clinically relevant animal models of sepsis [7]. Among immunomodulatory therapies, the blockade of PD-1/PD-L1 signaling could be a novel possible approach to reverse immunosuppression in sepsis, and upregulated expression of PD-1/PD-L1 in peripheral blood monocytes is observed in inflammatory diseases and cancers [8, 9]. But immunotherapeutic efficacy of anti-PD-1 antibodies on immune dysfunction of monocytes in sepsis remains unknown.

This work aimed to study PD-L1 expression on classical monocytes associated with sepsis severity and how PD-L1 blockade improves sepsis immune status by regulating monocyte function and provide evidence for sepsis immunotherapy.

## MATERIAL AND METHODS

### Subjects

**Fig. 1:**
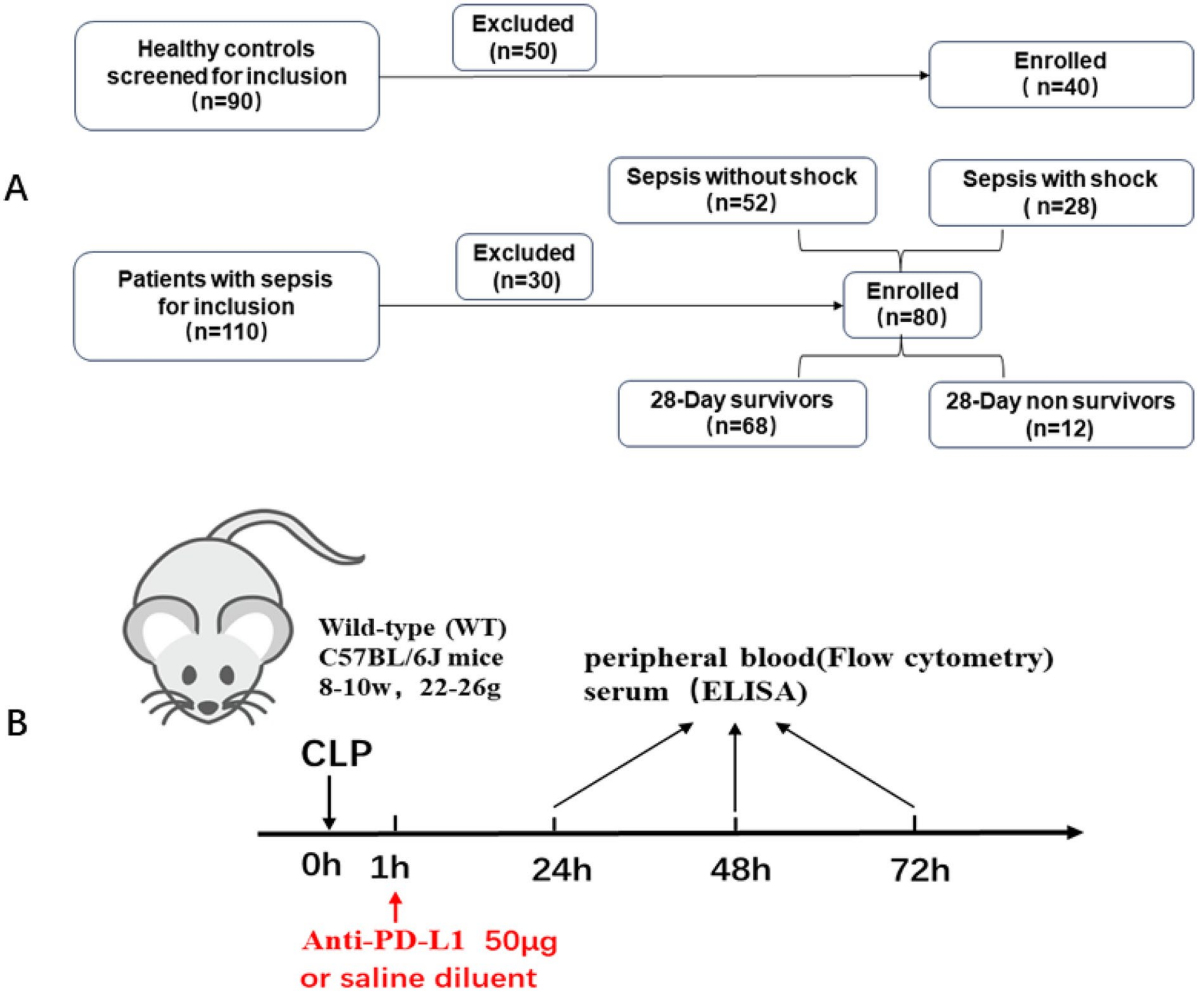
**A** Flow diagram of included patients and healthy controls. **B** Schematic diagram showing that WT mice were injected with PD-L1 antibody 1 h after CLP surgery and evaluated at 24, 48, and 72 h.

Eighty patients with sepsis were enrolled to emergency department (ED) of two hospitals (Beijing Shijitan Hospital and Beijing Chao-Yang Hospital), including 52 patients without septic shock and 28 patients with septic shock. In addition, 40 age- and sex-matched healthy volunteers were involved as the controls. Diagnosis of patient with sepsis was established according to the Third International Consensus Definitions for Sepsis and Septic Shock (Sepsis-3) [10]. Exclusion criteria were age < 18 years old, autoimmune disease history, infection in the last 6 months, cancers, pregnancy, and human immunodeficiency virus (HIV) infection history (Fig. 1A). We gathered peripheral blood samples (4 mL) within 24 h after confirming sepsis and summarized demographic and clinical symptoms of participants. Before performing this study, we have obtained written informed consent from all participants. This study was approved by the institutional ethics committees of the two aforementioned hospitals and conformed to the principles of Declaration of Helsinki.

### Animals

Wild-type (WT) male C57BL/6 J mice weighing approximately 22 g used in the study were acquired from the Jackson Laboratory (Sacramento, CA, USA). We housed all mice at standard laboratory conditions (20 ± 2 °C with 50 ± 5% relative humidity and a cycle of 12 h light with 12 h dark) and administered them food and water ad libitum but forbade them to eat and drink for 12 h before surgery.

### Cecal Ligation and Puncture Procedure

Cecal ligation and puncture (CLP)-induced polymicrobial sepsis was induced as described previously [11]. We anesthetized the mice using intraperitoneal injection of 2.5% tribromoethanol (0.02 mL/g; Sigma-Aldrich, St. Louis, MO, USA). After midline laparotomy, the cecum was exposed and ligated using nonabsorbable 4–0 silk distal to the ileocecal valve. We utilized 21-gauge needle to puncture cecal wall twice and extruded small stool (1 mm in length). Then, we performed the same procedure for sham-operated mice, like opening peritoneum and exposing the bowel, but with the lack of ligation or needle perforation of cecum. We resuscitated mice with subcutaneous injection of 1 mL of a sterile 0.9% saline solution after surgery and warmed the mice using heating lamp for 4 h for the purpose of increasing body temperature. We provided all the mice with unlimited food and water after surgery. We have obtained the prior consent from Animal Care and Use Committee of Capital Medical University (AEEI-2022–168) in terms of experimental procedures and conducted the experiments in accordance with Guidelines for Care and Use of Laboratory Animals made by US National Institutes of Health.

### Anti-PD-L1 Antibody Treatment Protocol

The anti-mouse PD-L1 monoclonal antibody was purchased from Bio X Cell (West Lebanon, NH, USA). About 50 μg/mouse anti-PD-1 antibody intraperitoneally was conducted on the mice at 1 h after CLP (Fig. 1B). We administered that anti-PD-L1 antibody dose was based on results from previous studies which demonstrated that 50 μg dose of anti-PD-L1 could play protective roles in cecal ligation and puncture (CLP) model of murine sepsis [12]. Mice from control group received sterile 0.9% saline solution.

### Flow Cytometry

We conducted analysis within 2 h when peripheral whole blood from patients with sepsis and normal control group, which were collected in ethylenediaminetetraacetic acid (EDTA) anticoagulant tubes. After lysing erythrocytes by lysing solution (BD Biosciences, San Jose, CA, USA), we stained cells by antibodies human CD45 (clone HI30), CD14 (clone M5E2), CD16 (clone 3G8), and CD274 (PD-L1), respectively, and evaluated using flow cytometry (Canto-II, BD). The antibodies were purchased from BD Biosciences (San Jose, CA, USA).

Mice were euthanized at 24, 48, and 72 h after CLP, and peripheral blood samples were obtained to analyze the expression of Ly6C^hi^ monocytes and MHC II on Ly6C^hi^ monocytes. Erythrocytes were lysed with lysis solution; then, the cells were stained with fluorochrome-conjugated anti-mouse CD14, anti-mouse CD11b, anti-mouse Ly6c, and anti-mouse MHC II (I-A/I-E). The antibodies were purchased from BioLegend (San Jose, CA, USA): PE anti-mouse CD14, Pacific Blue anti-mouse CD11b, PerCP anti-mouse Ly6c, and FITC anti-mouse I-A/I-E. Monoclonal antibodies and isotype controls were used according to the manufacturers’ recommendations. Samples were run on a BD FACSVerse flow cytometer (BD Biosciences) and analyzed using the FlowJo software (v. 10.0.8; Tree Star, Ashland, OR, USA). The results are expressed as percentages.

### Measurement of Serum Cytokine Levels

Sera from CLP- or sham-operated mice were collected at 24, 48, and 72 h after surgery and anti-PD-L1 treatment. The concentrations of TNF-α, IL-6, and IL-10 were detected by murine enzyme-linked immunosorbent assay (ELISA) kit (RayBiotech, Norcross, GA, USA), according to the manufacturer’s protocols. We conducted all tests at least thrice.

### Effect of PD-L1 Blockade on the Survival of Septic Mice

Wild-type mice were classified into the following: sham group, CLP-induced mice group, and CLP mice treated with anti-PD-L1 antibody group. For confirming protective effect anti-PD-L1 antibody on sepsis *in vivo*, we assessed survival rates over subsequent 7 days for three groups (*n* = 12 for each group). At 1 h after CLP surgery, we randomly injected C57BL/6 male mice intraperitoneally using anti-PD-L1 antibody (50 μg/mouse) and evaluated survival rate within 7 days. We administered all mice 1 mL of normal saline subcutaneously within half an hour after CLP and provided the mice with unlimited food and water.

### Statistical Analysis

All data are described as mean ± standard deviation (SD) or number (percentage). Normality test (Shapiro–Wilk test) was used for confirming normal distribution of the data. Student’s *t*-test was used to analyze the significance of the differences between two groups in normally distributed data, and Mann–Whitney *U* test was used for abnormally distributed data. The Kaplan–Meier method was conducted to analyze the survival rates after CLP operation and PD-L1 blockade. *P* < 0.05 was defined as statistically significant.

## RESULTS

### Patient Characteristics

The demographic and clinical characteristics of the patients are shown in Table 1. SOFA scores in the septic shock group were significantly higher than those in the non-septic shock group (*P* < 0.001) and proved that the SOFA scores are positively correlated with severity of sepsis in patients. The patients with septic shock got a higher 28-day mortality than those without shock. Compared to that in the septic group without shock, classical clinical inflammatory markers including C-reactive protein and procalcitonin were higher for patients with septic shock, but these trends were not significant in this work (*P* > 0.05). No statistically significant differences were observed in the total number of peripheral blood leukocytes or monocyte counts among groups (*P* > 0.05).

**Table 1:** The Demographic and Clinical Characteristics of Subjects

Variables	Healthy controls (*n* = 40)	Patients with sepsis (*n* = 80)		*P*
		Septic group (*n* = 52)	Septic shock group (*n* = 28)	
Age (year)	76 ± 5.75	81.10 ± 8.59	80.00 ± 8.38	0.085
Male (*n*, %)	24 (60)	26 (50)	17 (60.7)	0.530
WBC (10^9^ /L)	/	11.61 (7.53, 16.19)	10.86 (7.08, 17.95)	0.636
MO (10^9^ /L)	/	0.46 (0.33, 0.76)	0.44 (0.23, 0.61)	0.310
MOP (%)	/	4.50 (3.20, 6.10)	3.60 (2.13, 6.68)	0.338
CRP	/	110.59 ± 79.48	140.25 ± 94.52	0.194
PCT	/	11.50 ± 23.00	18.83 ± 40.44	0.476
SOFA score	/	5.33 ± 2.36	11.07 ± 1.39	< 0.001
28-day mortality (*n*, %)	/	5 (9.6)	7 (25)	0.003

Data shown are mean ± SD, median (Q1, Q3), and number (%). *P* < 0.05 was considered statistically significant

*WBC* white blood cell count, *MO* blood monocyte count, *MOP* blood monocyte percentage, *CRP* C-reactive protein, *ESR* erythrocyte sedimentation rate, *PCT* procalcitonin, *SOFA* sequential organ failure assessment

### Expression of PD-L1 on the MO1 Monocytes Among Different Groups

**Fig. 2:**
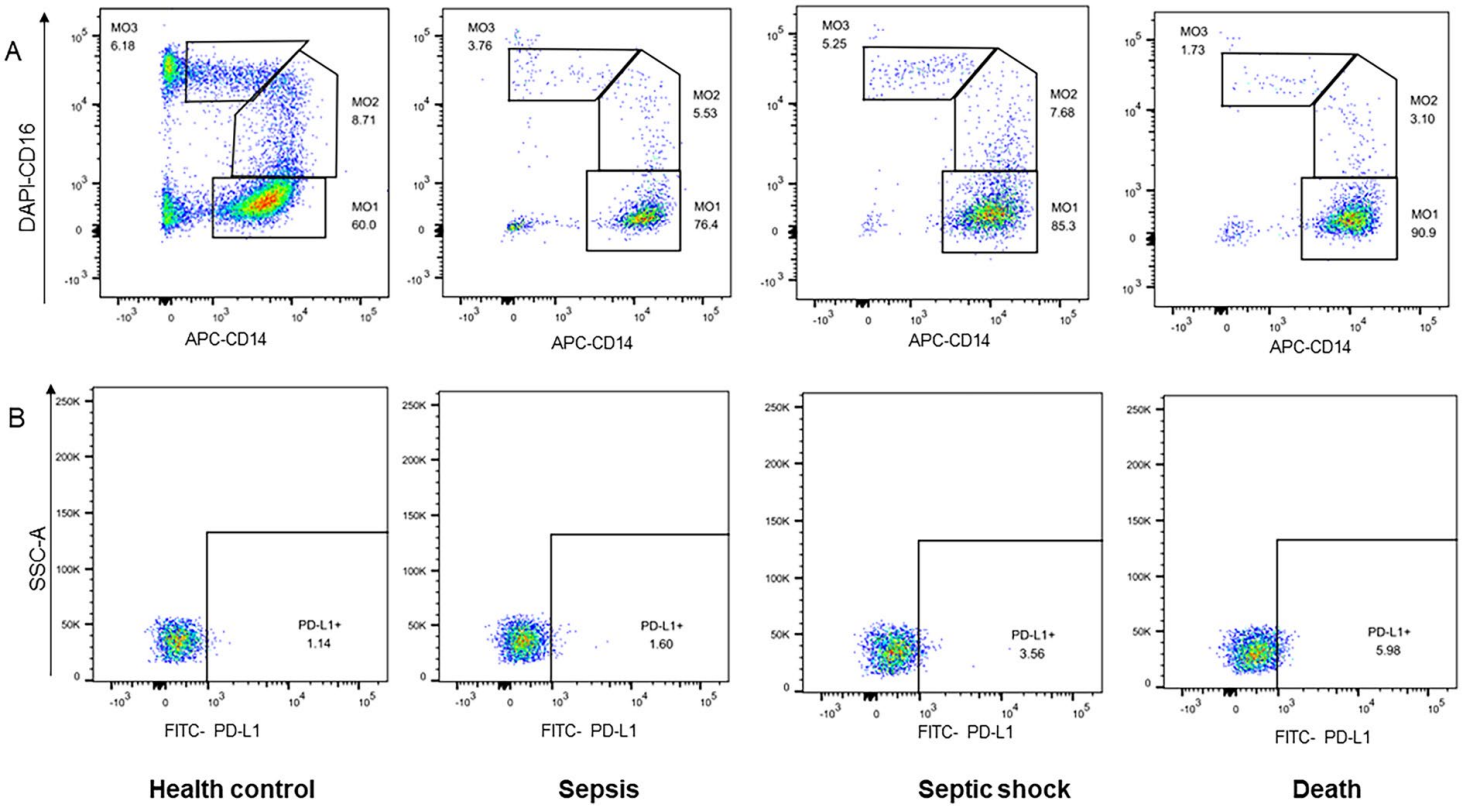
Flow cytometric detection-percentage of MO1 monocyte and PD-L1 on MO1 monocyte in health control, patient with sepsis, septic shock, and patients in 28-day death group. **A** The percentage of MO1 monocyte in each group. **B** The percentage of PD-L1 on MO1 monocyte in each group.

**Fig. 3:**
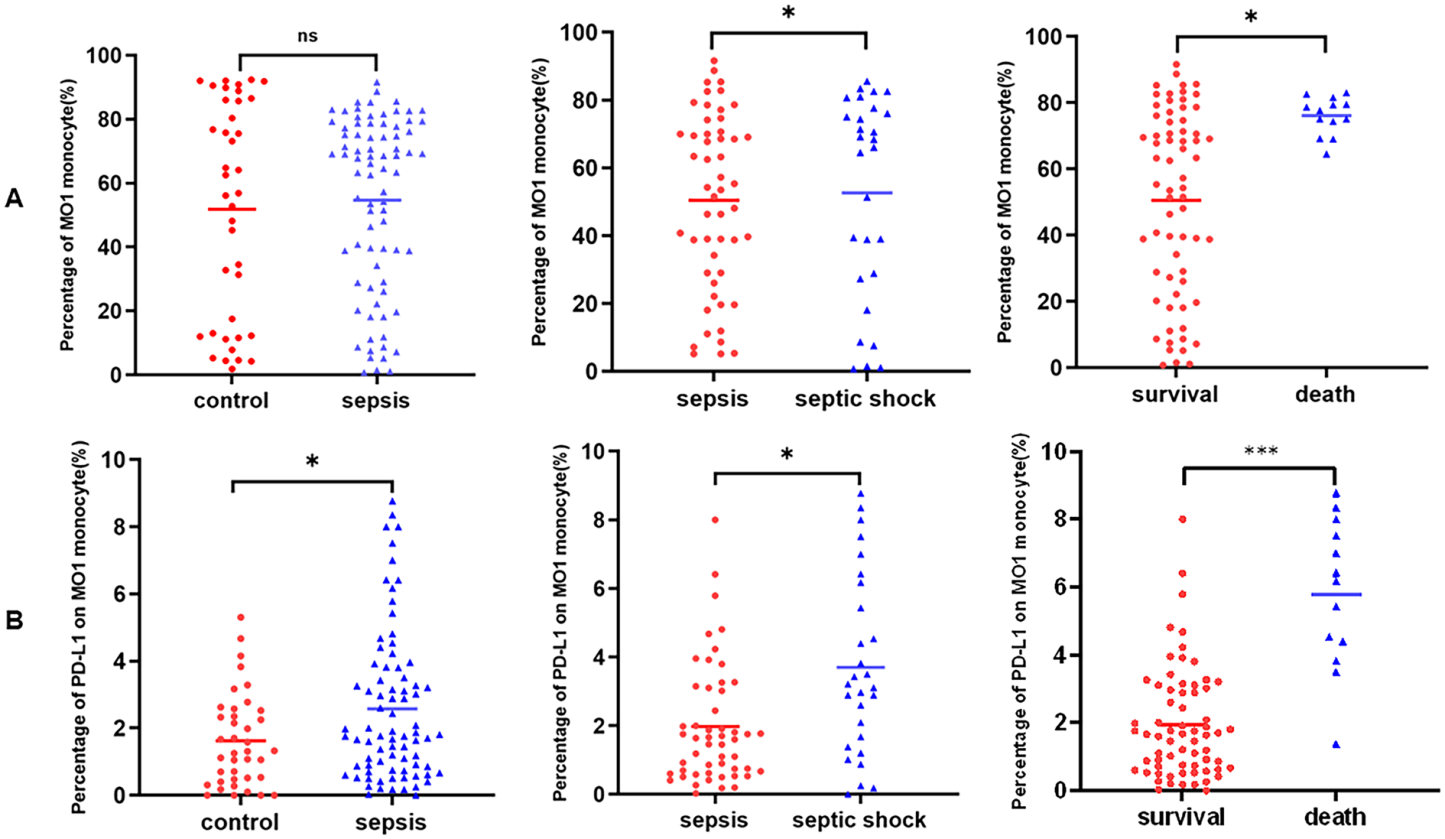
Comparing the percentage of MO1 (CD14 + + CD16 −) monocyte and PD-L1 on monocyte in peripheral blood between groups (control and patient, septic patients (no shock), and septic shock, patients in 28-day survival and death). **A** The percentage of MO1 (CD14 + + CD16 −) monocyte in three groups, respectively. **B** The percentage of PD-L1 on MO1 (CD14 + + CD16 −) monocyte in three groups. *P < 0.05; ***P < 0.001; ns, nonsignificant.

Monocytes in the peripheral blood can be divided into three subsets according to CD14 and CD16 expression: MO1 (classical, CD14 + + CD16 −), MO2 (intermediate, CD14 + + CD16 +), and MO3 (non-classical, CD14 + CD16 + +). Flow cytometric detection of the percentage of MO1 monocytes among groups is shown in Fig. 2A. We found that the percentage of MO1 monocyte was higher in septic shock patients than those without shock (septic group) (74.55% (68.15%, 79.35%) vs. 51.50% (21.20%, 70.65%), *P* = 0.006) and got conclusion from further analysis that percentage of MO1 monocytes in 28-day death group was dramatically higher than 28-day survival group (77.70% (74.40%, 79.50%) vs. 55.40% (26.70%, 74.45%), *P* = 0.002). However, no difference was observed in the percentage of MO1 monocytes between patients and controls (66.95% (36.15%, 78.15%) vs. 56.9% (12.65%, 85.85%), *P* = 0.930) (Fig. 3A).

Comparisons of PD-L1 expression on MO1 (CD14 + + CD16 −) monocytes among the groups are illustrated in Fig. 2B. We observed a significant upregulation of PD-L1 expression on MO1 (CD14 + + CD16 −) monocytes in patients with septic compared to the control group (1.79% (0.80%, 3.65%) vs. 1.31% (0.50%, 2.44%), *P* = 0.032). Moreover, PD-L1 expression on MO1 (CD14 + + CD16 −) monocytes was significantly higher in patients with septic shock and 28-day death group than that in patients without shock group and 28-day survival group (3.07% (1.53%, 5.12%) vs. 1.66% (0.61%, 3.12%), *P* = 0.008; 6.17% (4.40%, 7.51%) vs. 1.66% (0.69%, 2.99%), *P* < 0.001) (Fig. 3B). In addition, no difference was observed in PD-L1 expression on MO2 and MO3 monocytes among aforementioned groups (*P* > 0.05). Our data showed that PDL-1 expression on MO1 (classical monocytes) was deeply involved in severity and prognosis of sepsis.

### Blockade of PD-L1 Signaling Pathway Effect on Percentage of Ly6C^hi^ Monocytes in CLP Mice

**Fig. 4:**
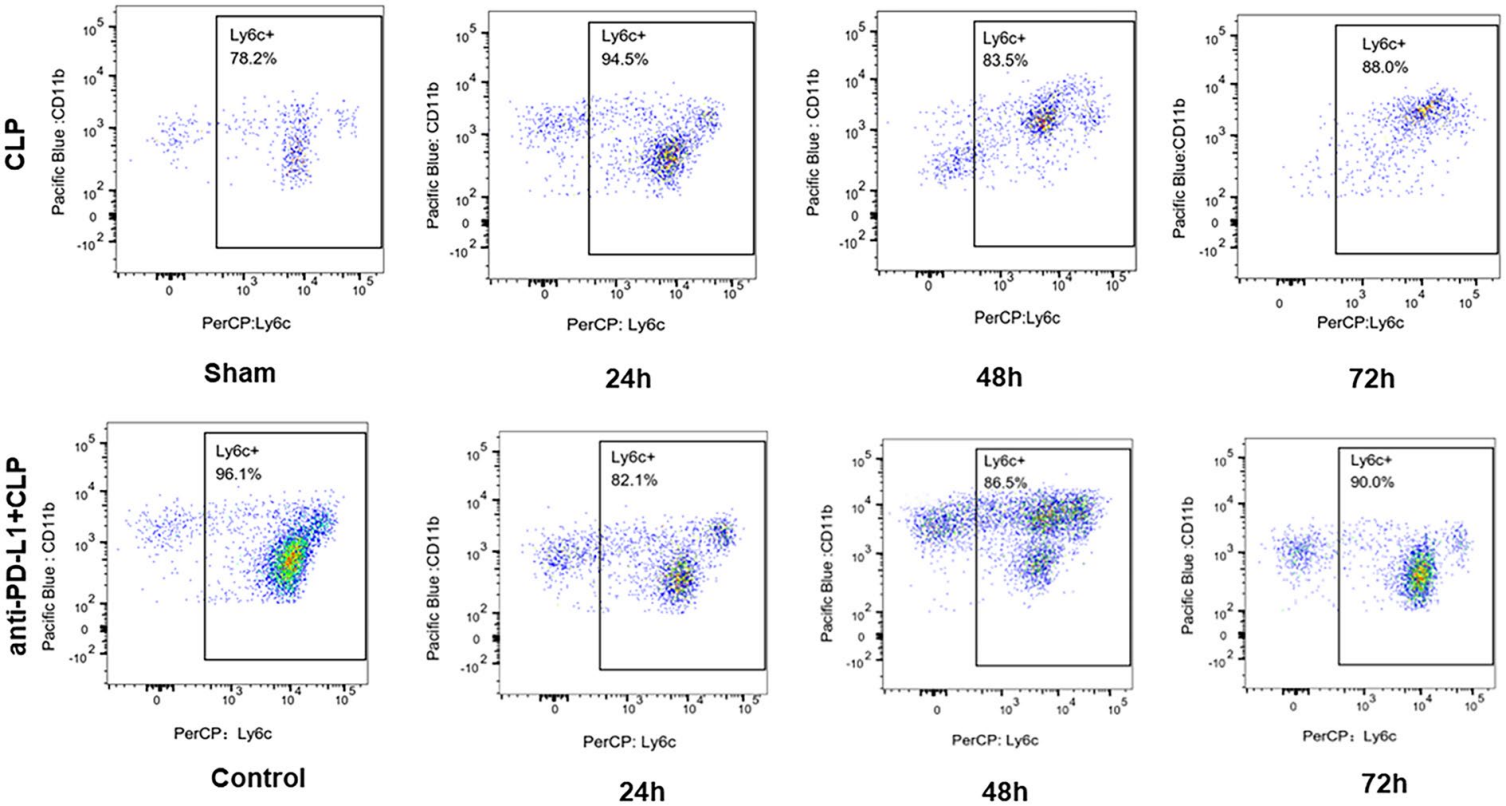
Flow cytometric detection-percentage of Ly6C^hi^ monocyte in peripheral blood in CLP mice and PD-L1 blockade CLP mice at control, 24, 48, and 72 h.

**Fig. 5:**
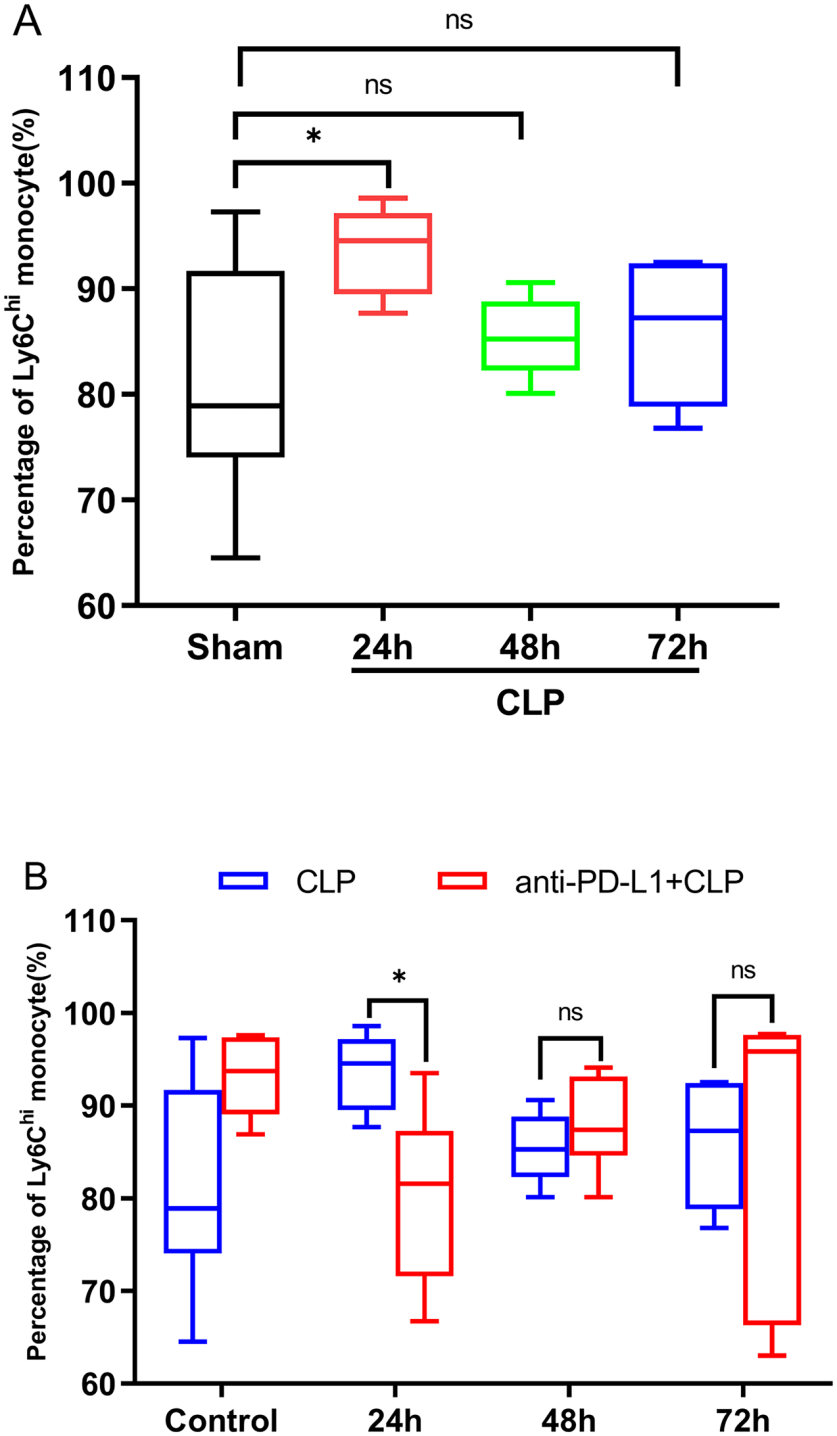
Effects of the PD-L1 blocked on percentage of Ly6C^hi^ monocyte in peripheral blood of CLP-operated mice in different groups. **A** Percentage of Ly6C^hi^ monocyte in the peripheral blood of mice at 24, 48, and72 h after CLP surgery (*n* = 6). **B** Percentage of Ly6C^hi^ monocyte in the peripheral blood of CLP-treated mice after PD-L1 blockade in three groups (*n* = 6). The data analysis is conducted by Mann–Whitney test, and *n* represents the number of mice in each group *P < 0.05; ***P < 0.001; ns, nonsignificant.

To elucidate the impact of the anti-PD-L1 antibody on the proportion of classical monocytes in the CLP-induced sepsis model, we measured the percentage of Ly6C^hi^ monocyte at 24, 48, and 72 h and sham group. Flow cytometry (Fig. 4) indicated a significant increase in Ly6C^hi^ monocyte proportion in the peripheral blood of CLP-induced septic at 24 h (*P* = 0.028), followed by a downward trend at 48 and 72 h compared to the sham group; however, no significant differences were observed (Fig. 5A). Anti-PD-L1 antibody changed the Ly6C^hi^ monocyte ratio in mice after the CLP operation at an early stage (24 h, *P* = 0.016); however, there were no statistical differences at 48 and 72 h (*P* > 0.05) (Fig. 5B).

### Blockade of the PD-L1 Signaling Pathway Triggered the Percentage Increase of MCH II Levels on Ly6C^hi^ Monocytes in CLP Mice

**Fig. 6:**
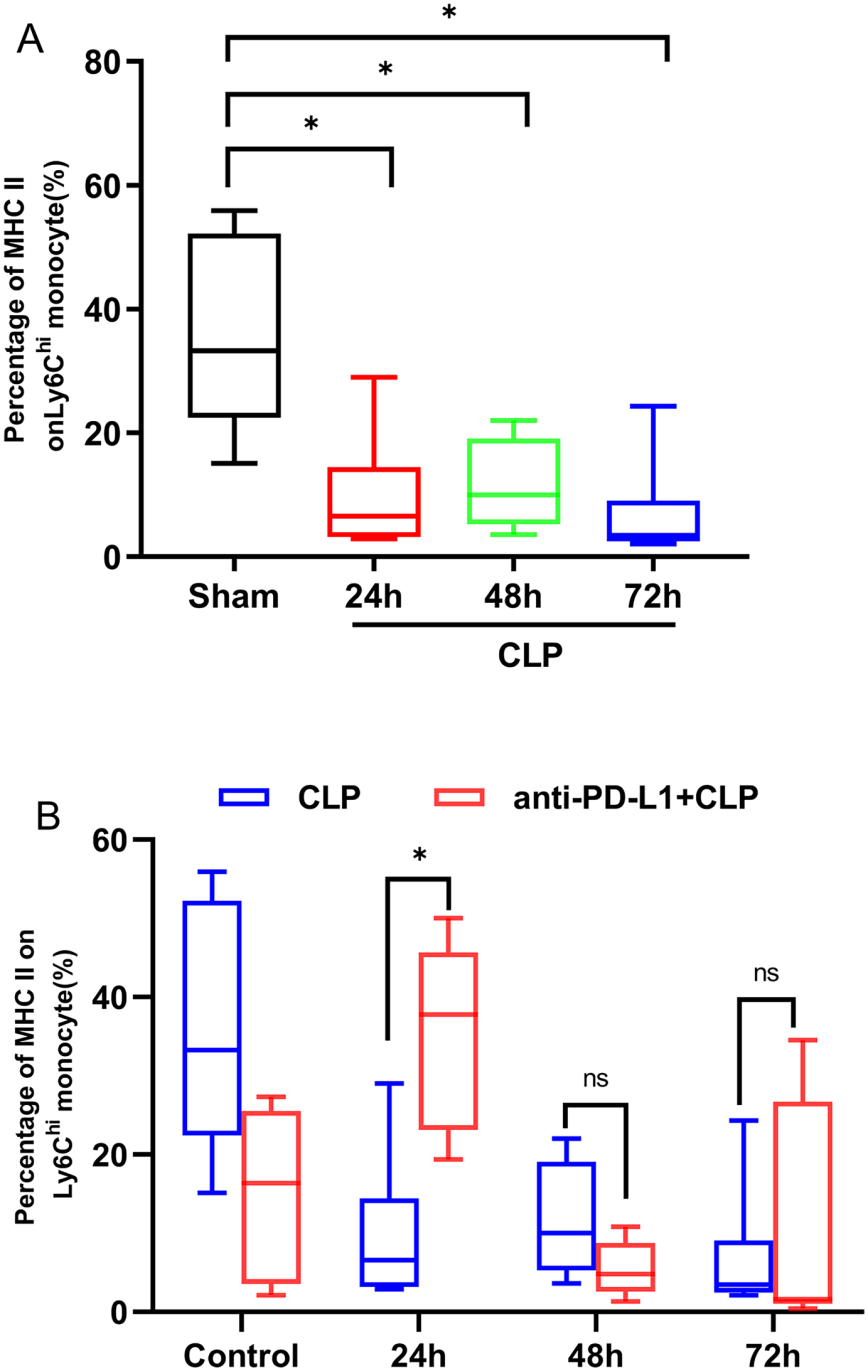
Effects of PD-L1 blocked on percentage of MHC II on Ly6C^hi^ monocyte in the peripheral blood of CLP-operated mice at 24, 48, and 72 h. **A** Percentage of MHC II on Ly6C^hi^ monocyte in the peripheral blood of CLP-treated mice at 24, 48, and 72 h (*n* = 6).** B** Percentage of MHC II on Ly6C^hi^ monocyte in the peripheral blood of CLP-treated mice after PD-L1 blockade at three time points (*n* = 6). Data analysis is conducted by Mann–Whitney test, and *n* represents the number of mice in each group. *P < 0.05; ***P < 0.001; ns, nonsignificant.

To evaluate sepsis-induced monocyte activity, we examined MHC II expression in monocytes. MHC II expression in monocytes decreases during sepsis. Here, we used flow cytometry to quantify the effects of an anti-PDL1 antibody on MHC II expression in peripheral Ly6C^hi^ monocytes of mice after CLP. Our data revealed that sepsis caused an evident decrease in the percentage of MHC II expression in peripheral Ly6C^hi^ monocyte at 24, 48, and 72 h compared to that in the sham group (*P* = 0.016, *P* = 0.012, *P* = 0.004) (Fig. 6A). Compared to septic mice, anti-PD-L1 antibody-treated mice had significantly elevated percentages of MHC II in peripheral Ly6C^hi^ monocyte at 24 h (*P* = 0.009); however, no statistical difference was observed at 48 h and 72 h points (*P* > 0.05) (Fig. 6B). Our data demonstrate that anti-PD-L1 antibodies have an effect on improving MHC II expression in peripheral Ly6C^hi^ monocytes in mice with sepsis during the early disease course. Our research showed that anti-PD-L1 antibodies can improve monocyte function in the early stages of sepsis.

### Blockade of the PD-L1 Signaling Pathway Reduces Secretion of Inflammatory Cytokines in CLP Mouse Serum

**Fig. 7:**
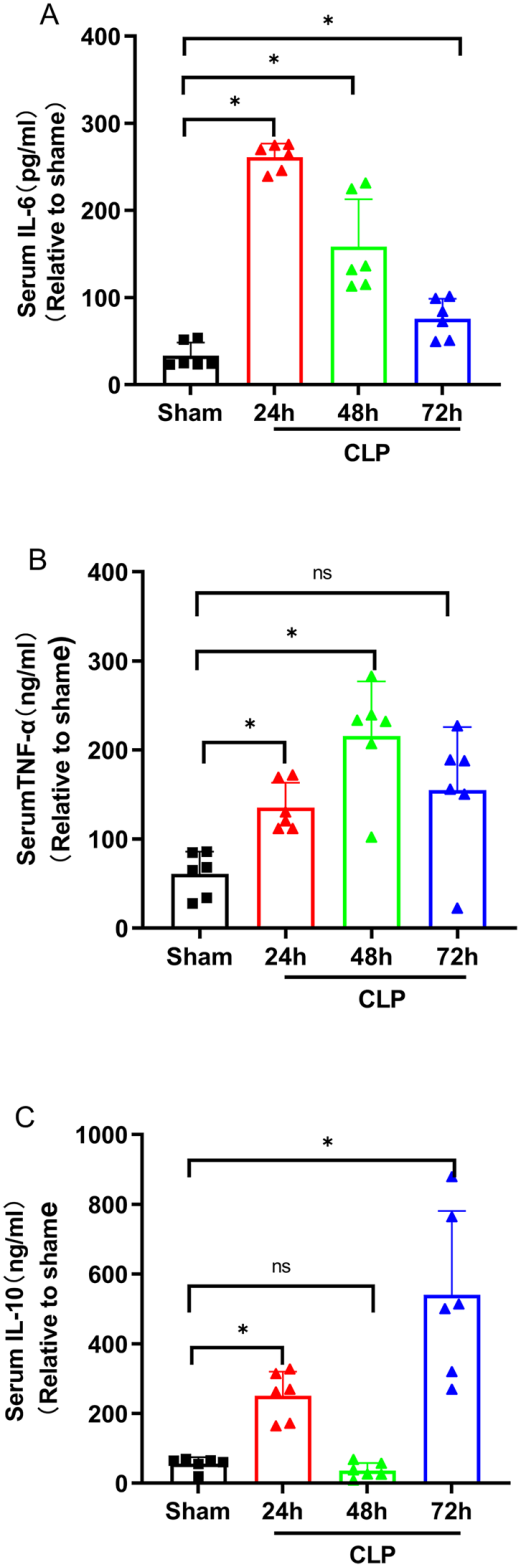
Expression levels of IL-6, IL-10, and TNF-α in serum of mice at 24, 48, and 72 h after CLP surgery and sham control. **A** Expression of IL-6 in CLP-treated mouse serum at three time points (*n* = 6). **B** Level of serum TNF-α in CLP-operated mice at three time points (*n* = 6). **C** Expression of serum IL-10 after CLP operation mice at three time points (*n* = 6). The data are shown as the mean ± SD, and *n* represents the number of mice in each group. *P < 0.05; ***P < 0.001; ns, nonsignificant.

**Fig. 8:**
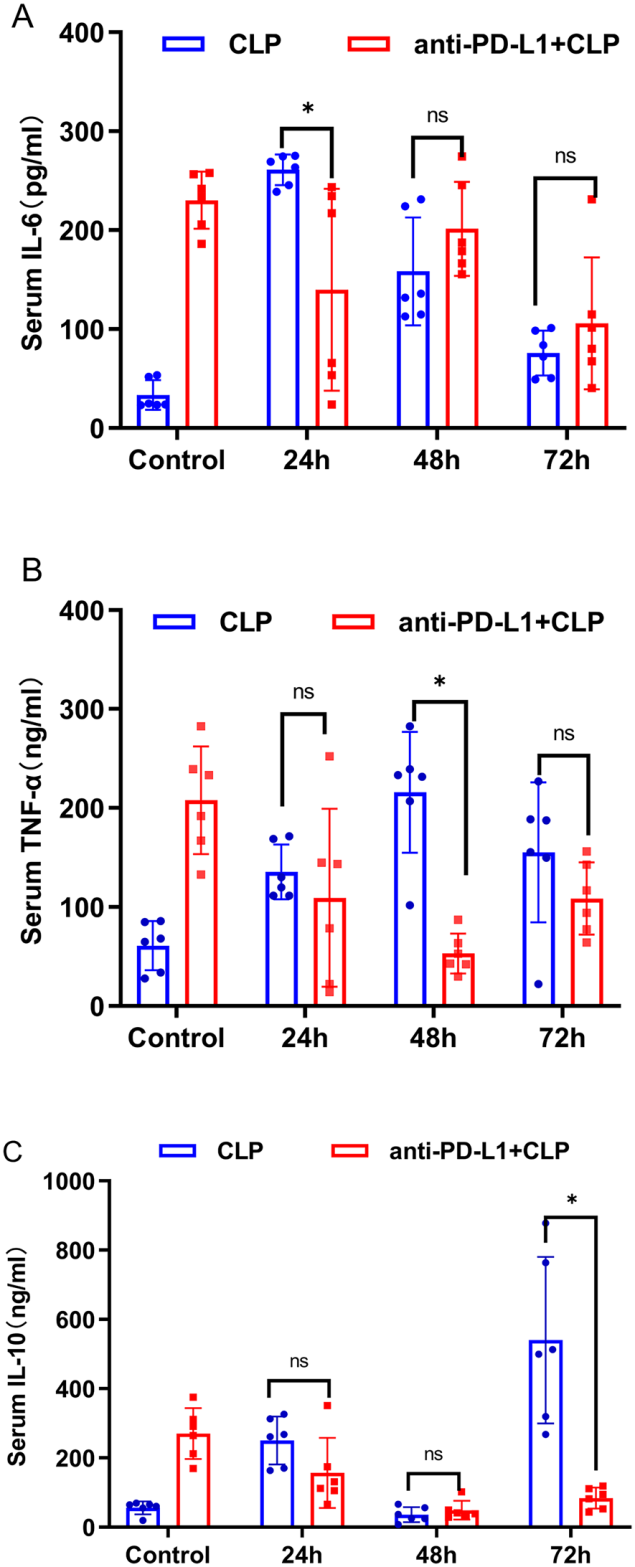
Effects of anti-PD-L1 antibody on the serum cytokines in CLP-induced sepsis mice at 24, 48, and 72 h and sham control. **A** Expression of IL-6 in CLP-treated mice serum after PD-L1 blockade at three time points (*n* = 6).** B** Level of serum TNF-α in CLP-operated mice after PD-L1 blockade at three time points (*n* = 6). **C** Expression of IL-10 in serum of CLP-treated mice after PD-L1 blockade at three time points (*n* = 6). The data are shown as the mean ± SD, and *n* represents the number of mice in each group. *P < 0.05; ***P < 0.001; ns, nonsignificant.

Major secretions of monocytes are IL-6, IL-8, IL-10, TNF-α, MCP-1, and IL-1β [13]. To investigate whether the anti-PD-L1 antibody affected monocyte function, we selected three important cytokines, IL-6, IL-10, and TNF-α, used ELISA to assess cytokine production in the CLP-induced sepsis model and anti-PD-L1 antibody-treated septic mice at 24, 48, and 72 h. We found that the levels of IL-6 (Fig. 7A), TNF-α (Fig. 7B), and IL-10 (Fig. 7C) in the serum were upregulated in CLP-induced sepsis mice than those in sham group. Serum IL-6 levels in septic mice peaked at 24 h (*P* = 0.002), which defined the hyperinflammatory state, and with a declining trend at 48 and 72 h (*P* < 0.05). The level of serum TNF-α increased gradually with time, reached a peak at 48 h (*P* = 0.002) after the CLP operation, and then decreased at 72 h point (*P* > 0.05). Serum IL-10 level in CLP-induced sepsis mice showed an increasing trend and reached a peak at 72 h (*P* = 0.002). Our data demonstrated that PD-L1 blockade reduced the secretion of inflammatory cytokines in mice with CLP-induced sepsis. Levels of serum IL-6 (Fig. 8A), TNF-α (Fig. 8B), and IL-10 (Fig. 8C) in sepsis mice treated with anti-PD-L1 antibody significantly declined at 24 (*P* = 0.004), 48 (*P* = 0.006), and 72 h (*P* = 0.005), respectively; there were no significant changes at other time points, showing no statistical significance. Our research observed the CLP-induced production of inflammatory cytokines, which was attenuated by PD-L1 blockade at different points in time.

### Blockade of the PD-L1 Improved the Survival Rate of Sepsis Mice

**Fig. 9:**
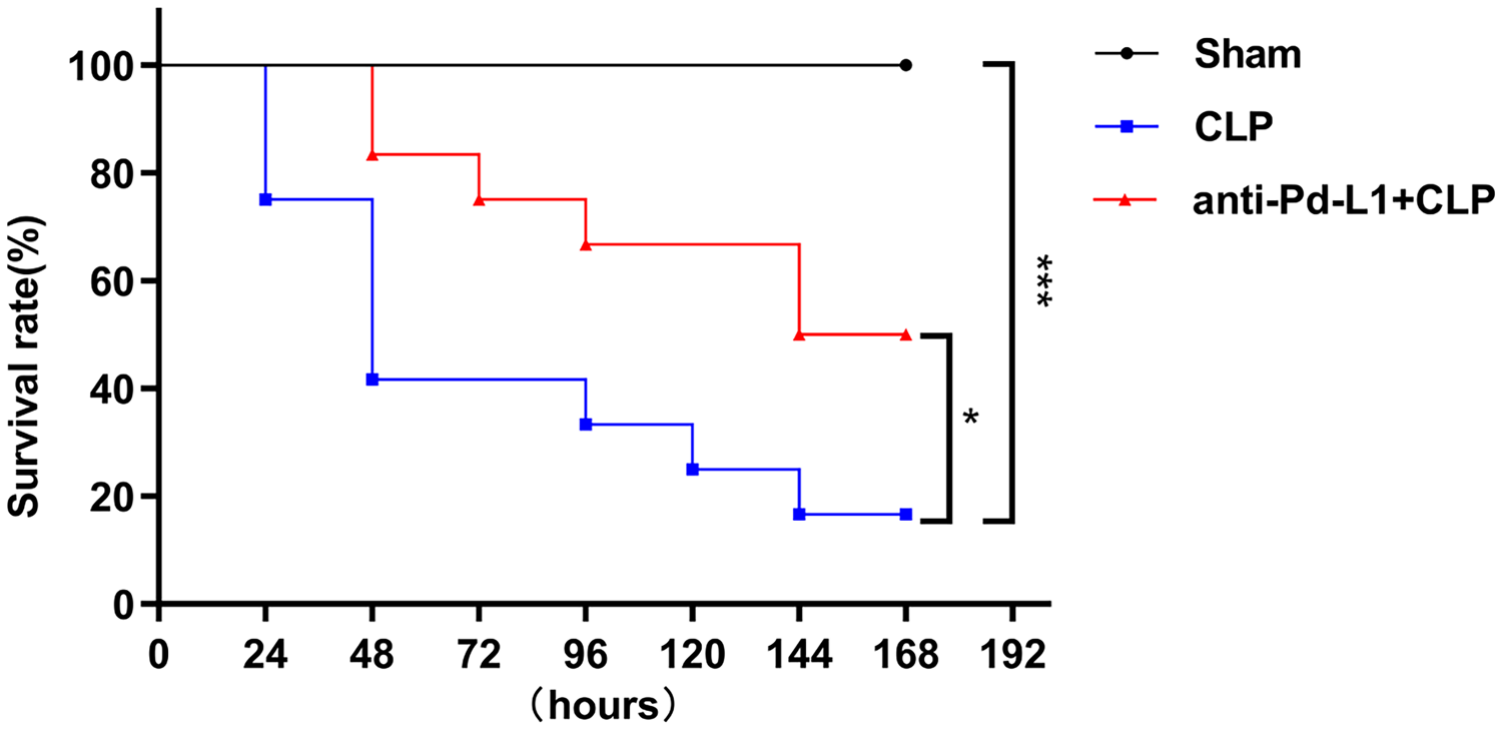
Survival rate of mice for 7 days in each group (*n* = 12). The difference in survival rate among groups was compared using Kaplan–Meier survival analysis, and *n* represents the number of mice in each group. *P < 0.05; ***P < 0.001.

We used Kaplan–Meier method to analyze the 7-day survival rate of mice in three groups (Fig. 9). Our data revealed that the survival rate of mice after CLP operation decreased significantly compared to the sham group (*P* = 0.001). The survival rate of mice treated with anti-PD-L1 antibody at 1 h after CLP was higher (50.0%) than that of CLP-operated mice (16.7%; *P* = 0.035). After CLP, the administration of anti-PD-L1 antibody dramatically decreased animal lethality, attributed to the amelioration of systemic inflammation (flow cytometry and ELISA).

## DISCUSSION

The primary immune events in sepsis are high inflammation and immune failure (or immune paralysis) caused by cytokine storms, both of which may occur simultaneously or asynchronously [5], which may eventually result in death. Early diagnosis and appropriate management of sepsis play a critical role in preventing life-threatening complications. Innate immune cells represent the first line of defense after infection and, thus, play a central role in controlling pathogens and initiating adaptive immune responses. Monocytes are immune cells with roles in cytokine production, antigen presentation, and phagocytosis. Recently, monocytes have been classified into three subsets based on the expression of CD16 and CD14, classical monocytes (CD14 + + CD16 −), and non-classical monocytes (CD14 + CD16 + +), which accounts for approximately 90% and 5% circulating monocytes, respectively, and intermediate monocytes (CD14 + + CD16 +) [14, 15]. Classical monocytes were closely correlated with immune responses, such as phagocytosis, endothelial transmigration, and secrete inflammatory cytokines [13], and its number significantly increases in patients with sepsis [16, 17]. In mice, ‘‘classical’’ monocytes are characterized by the surface marker Ly6C^hi^ (previously termed inflammatory monocytes), whereas ‘‘non-classical” monocytes (also termed patrolling monocytes) are defined as Ly6C^low^, corresponding to human “classical” CD14 + + CD16 − monocytes and “non-classical” CD14 + CD16 + + monocyte, respectively [18]. Disparate monocyte subsets exhibit distinct immune effects and perform diverse functions, characterized by differences in inflammatory factor release, phagocytosis, and antigen presentation. Monocyte dysfunction is an important characteristic of immune functional imbalance in sepsis. Many studies have proofed correlation between low levels of monocyte HLA-DR (a member of the MHC II family) and impaired monocyte function [19, 20]. Most importantly, decreased MHC II expression is a sign of immunosuppression, and its persistent decrease is closely related to the severity and mortality of patients with sepsis [21, 22], and the slower the recovery, the higher the risk of secondary infection [23]. Therefore, HLA-DR expression is thought to serve as a biomarker for identifying patients for immunotherapy and monitoring responsiveness to treatment [24]. Our data showed that the proportion of classical monocytes elevated significantly in patients with septic shock and death, and in mice with sepsis, classical monocytes increased significantly at 24 h. The increased expression of classical monocytes is closely associated with severity and prognosis of sepsis. Our study also demonstrated the reduction of MHC II on monocyte at 24 h post-CLP mice, indicating an immunosuppressive state.

The PD-1/PD-L1 signaling pathway plays a critical role in autoimmune diseases, infectious diseases, tumor immunity, and drug resistance mechanisms [25–28]. Given the remarkable success of tumor immunotherapy, similar immune deficiencies in sepsis and cancer, and the high mortality rate of patients with sepsis, therapeutic trials to improve host immunity ought to be top priority. Several studies have reported upregulated PD-L1 expression in various cell types during sepsis. Patients with sepsis, especially those with severe sepsis and septic shock, have significantly elevated levels of PD-1 expression in CD4 + or CD8 + T cells and PD-L1 expression in monocytes [29, 30]. The PD-1/PD-L1 signaling pathway is involved in immunosuppression and organ damage in sepsis. Clinical trials have demonstrated that PD-L1 expression on the surface of peripheral blood monocytes in septic shock patients is increased [31]. Several studies have reported that significantly increased PD-L1 levels in monocytes are closely correlated with immunosuppression in patients with sepsis and acute pancreatitis with infectious complications [32, 33]. This indicates that PD-L1 is associated with the immune regulation in sepsis. Our data showed that PD-L1 on MO1 monocytes was remarkably increased in patients with septic shock and the death group compared to that in patients without shock and the survival group. Increased PD-L1 expression in monocytes of patients had relation to severity and mortality in sepsis, which is consistent with the results of Shao et al*.*’s research [34]. The results suggest a positive correlation between the severity of sepsis and the expression levels of PD-L1. Increased PD-L1 levels possibly imply immune dysfunction of patients with severe sepsis or septic shock; thus, it is reasonable to speculate that PD-L1 possibly has relation to monocyte regulation of patients with severe sepsis, particularly MO1 monocyte regulations which are closely correlated with inflammation. Our analysis highlights the importance of PD1/PD-L1 on monocytes in immunoregulatory system during sepsis-induced immune alterations. Therefore, blocking PD1/PD-L1 pathway can alter immune status of patients with sepsis and improve their survival rate.

Inflammatory cytokines are commonly regarded as the primary indicators of sepsis pathophysiology. Several studies have shown that monocytes secrete fewer cytokines during sepsis-induced immunosuppression. Experimental studies have demonstrated that increased PD-1/PD-L1 expression on surface of peripheral blood monocytes or macrophages in mice with sepsis may lead to a deterioration in the phagocytic functions, downregulate release of pro-inflammatory cytokines, like IL-6 and TNF-α, and upregulate release of anti-inflammatory cytokines, like IL-10 [12, 35]. Consistent with recent findings, we observed that CLP-induced sepsis led to cytokine production in a time-dependent manner. Our study found that the level of serum IL-6 and TNF-α of sepsis mice was observed greatly upregulated at hyperinflammatory state and then dramatically downregulated at immunosuppressive state. In contrast, the level of immunosuppressive cytokine IL-10 was markedly increased. Based on the aforementioned findings, we confirmed that alterations in immune cells and cytokines in peripheral blood are associated with immune disorder sepsis. Our research found that after anti-PD-L1 antibody treatment, inflammatory monocytes downregulated and MHC II upregulated significantly at the 24 h point; additionally, CLP-induced production of inflammatory cytokines was attenuated at different points in time. This finding suggested that anti-PD-L1 may play a crucial role in both early and later stages in the CLP-induced sepsis. Our findings on IL-6 levels are contrast with some prior research showing that blockade of the PD-1/PD-L1 interaction enhances IL-6 production during sepsis [12, 36], whereas we observed that anti-PD-L1 antibody suppressed both pro-inflammatory cytokines (IL-6 and TNF-α) and anti-inflammatory cytokines (IL-10) in this work, which is consistent with previous reports [37, 38]. Administration of anti-PD-L1 antibodies prevented sepsis-induced monocyte dysfunction and decreased TNF-α, IL-6, and IL-10 production at different stages of sepsis, implying an overall reduction in systemic inflammation. The current results are consistent with those of previous studies showing that blocking or knocking out PD-1/PD-L1 signaling pathway can reverse immune cell dysfunction, affect the secretion of inflammatory cytokines, and improve survival in sepsis [32, 36, 39, 40].

We confirmed that anti-PD-L1 administered 1 h after CLP caused significant upregulation of MHC II in MO1 monocytes, improved monocyte function, reduced the production of inflammatory cytokines, prevented immune suppression, and enhanced survival in CLP mice. Survival improvement should be a crucial evaluation metric for all treatments. The present findings demonstrate that blocking the PD-1/PD-L1 pathway plays a protective effect role in sepsis mice. Thus, the attenuation of monocyte-mediated inflammatory damage can be a promising therapy for sepsis. However, our study is subject to certain limitations. Firstly, due to the limited sample size in this research, certain results may not accurately reflect the actual situation; it is needed to increase the sample size in future studies. Second, we shall make great efforts on anti-PD-L1 antibodies which investigate at different time points and doses in sepsis models.

Although murine models cannot be extrapolated directly to clinical treatments, they can help evaluate pathogenic mechanisms potentially contributing to human disease. PD-L1 is crucial to immune response regulation of monocytes in patients with sepsis. In the near future, this molecule may become one of targets for immunotherapy of sepsis.

## CONCLUSIONS

Our research suggests that classical monocytes with high expression of PD-L1 are associated with the progression of sepsis. The anti-PD-L1 antibody altered systemic cytokine release, affected the function of monocytes, and reduced the mortality of mice with sepsis, suggesting that monocytes could be potential therapeutic targets in sepsis.

## Data Availability

The data set generated during this study is available from the corresponding author on reasonable request.
